# Robotics in Arthroplasty: Historical Progression, Contemporary Applications, and Future Horizons With Artificial Intelligence (AI) Integration

**DOI:** 10.7759/cureus.67611

**Published:** 2024-08-23

**Authors:** Jagbir Singh, Priyankkumar Patel

**Affiliations:** 1 Robotics, Smith and Nephew, Pittsburgh, USA; 2 Quality Engineering, Smith and Nephew, Pittsburgh, USA

**Keywords:** tka, tha, robotic, orthopedic, machine learning, artificial intelligence

## Abstract

Robotic technology is increasingly utilized in surgical procedures to enhance precision, particularly in tasks demanding delicate maneuvers beyond human capabilities. Robotic orthopedic surgery emerges as a dynamic and compelling technology reshaping the landscape of surgical practice. This aids surgeons in achieving enhanced accuracy and reproducibility, ultimately aiming for improved patient outcomes. As of now, the majority of these systems are in a developed stage and are gradually gaining broader adoption. These systems have to show that they are user-friendly, are successful in clinical settings, and have a good cost-effectiveness ratio before they can be widely adopted in the field of surgery. In this review, we examine the evolution of robotics in orthopedic surgery, assess its current applications, and provide insights into the future trajectory of this technology, particularly in light of advances in artificial intelligence (AI) and machine learning (ML).

## Introduction and background

In his play "Rossum's Universal Robots", written in 1921, Czech playwright Karel Čapek invented the term "robot" and its meaning [1]. The idea of robots initially acquired importance among people, but recent advances in manufacturing, mechanical, electrical, and electronic technology, along with a sharp increase in microcontroller power, have made robots more than just concepts. With a high-speed microcontroller, the motor controller algorithm can be executed in thousands of seconds or less. Despite this, the adoption of robotics in the medical sector has been relatively sluggish. George Devol created the initial robotic device, the "Unimate" hydraulic programmed manipulator, in the late 1950s [2]. Joseph Engelberger later purchased this device for use in the industry in the 1960s. Because of his work and accomplishments, Joseph Engelberger is referred to in the field as "the Father of Robotics" [3]. Unimation, the planet's first robotics organization, was formed by Devol and Engelberger [4]. Based on the works of Devol and Engelberger, the business created the first Unimate robot, which it sold to General Motors in 1960 so that it could be utilized to stack and carry hot metal pieces. These advancements achieved in industrial robotics paved the way for significant progress in surgical technology in the 1980s. In terms of surgical robotics, the field began to use robotic technology in the middle of the 1980s after a system specifically designed for stereotactic neurosurgery was created. For the purpose of doing neurosurgical biopsies, the Puma 560 robotic surgical system was originally presented in 1985 by Armstrong et al. [5]. By using computed tomography (CT) guidance, this robot improved precision during neurosurgical biopsies. In 1988, its use was broadened to include prostate transurethral resection, which set the foundation for the Probot system by Harris et al. [6]. This unique apparatus, designed to make prostatic tissue removal easier, proved that robotics might be used in soft tissue surgery. Developed by William Bargar, ROBODOC was a ground-breaking active robotic system designed exclusively for orthopedics [7]. Originally intended for cementless total hip arthroplasty (THA), the ROBODOC's uses were eventually broadened to encompass revision THA and primary total knee arthroplasty (TKA). The ROBODOC was performing surgeries worldwide but received an FDA approval in August 2008. A commercial robotic surgical system called CASPAR by Ortho-Maquet/URS, Schwerin, Germany, was modified to help the surgeon with TKR intraoperative execution and preoperative planning [8]. Competing against one another at the time were CASPAR and ROBODOC, which represented the innovative combination of two newly developing technologies: robotics and medical imaging. Both were initially developed as CT-based, computer-aided robotic milling devices, enabling the precise preparation of the femoral bone and anatomically correct placement of the femoral component in cementless THA. The company behind ROBODOC changed ownership, but the company behind the CASPAR robotic system is no longer in business. The MAKO [9] robotic arm-supported device cleared the FDA in early 2008 and offered further improvements in arthroplasty procedures. This technology lets the doctors to see the joint, alignment, and abnormalities more clearly before surgery by using preoperative CT images. This three-dimensional preoperative image virtually balances the knee ligaments and allows for implant locations to be customized to the patient's anatomy. As a result, more businesses are joining the robotic arthroplasty industry, giving surgeons a wide range of alternatives to choose from. However, the earlier robotic systems, like ROBODOC, failed to gain widespread surgeon acceptance as the surgeon was not actively involved. Acrobot and Rio are two examples of semi-active robotic technologies designed to make surgery easier for surgeons to accept [10,11]. With these robotic systems, a surgeon's hand moves the drill bit at the tip of the robotic arm, but it stays inside the milling route boundary, which is determined by three-dimensional image-based preoperative planning. Traditional arthroplasty is widely acknowledged as a safe and economical treatment solution for osteoarthritis. However, patient satisfaction rates for these procedures remain a concern. The study is as follows: Firstly, we will see the surgical robot classification. Secondly, the challenges are presented. Thirdly, the artificial intelligence (AI) in surgical robotics is discussed. The discussion section is presented next, and then finally we conclude the study with some conclusion and future works.

Surgical robot classification

Based on Methods to Robotic Arthroplasty Surgery: Active, Semi-active, and Passive

Robotic surgery uses a variety of robot types, enabling a range of execution techniques. Three primary categories have been established for these robotic approaches: semi-active, active, and passive. "Passive systems" are those that don't do anything during an operation, but instead offer more information. Part of the procedure is completed by passive systems, which are continuously and directly controlled by the surgeon. In TKA, computer navigation is the most common kind of passive computer-assisted surgery (CAS). The passive CAS plays a supporting role to the surgeon, who maintains direct control throughout the process. Feedback on the orientation, range of motion (ROM), and alignment of the bony cuts can be obtained through navigation. Following a boom in the 1990s, the technology's appeal declined, and many businesses gave up on it due to the additional expense and lack of therapeutic benefit. Although semi-active systems need the surgeon to be involved, they can theoretically increase operative safety and control by giving tactile input. "Haptic systems" is another term for these semi-active systems. Surgical resection is performed using passive haptic restraints by the robotic system, as opposed to passive guidance and feedback by CAS. As a result, the surgeon is limited to treating the intended amount of resection in three dimensions and cannot, for example, burr bone outside of the predetermined volumetric constraints. The surgeon receives haptic feedback via tactile (vibratory), visual (color changes on the computer screen), and aural (beeping). As the specified resection parameters are approached, these alarms start to sound, giving the surgeon feedback and preventing over-resection and improper placement throughout the procedure. Clinical decision-making is facilitated by the process, which depends more on quantitative data than on the senses and intuition of the surgeon. A different kind of semi-active technology regulates the functioning instrument's depth and speed. The semi-active system connects the burr's placement within the area of operation once a well-defined bone resection program has been prepared and the process has commenced. The computer system will either slow down the burr's pace or retract it into the handpiece once it gets close to the edge of the intended resection, hence reducing the chance of over-resectioning the bone. Through feedback and controls that reduce mistakes and increase precision, this technology enables the surgeon to accomplish the bone excision within predetermined parameters. Active systems, like CASPAR and ROBODOC, can carry out a task without the need for a surgeon to be involved [12].

Based on Classification on Imaging Modality Used: Image-Based Versus Imageless Robotics Solution

Currently, a platform and "preapproved" plan is needed for any orthopedic robotic system in order to start the surgical operation. These systems can have an image or not have an image. In order for the "robot" to know where the cutting tools are with respect to the anatomy, the anatomy of the patient must be recorded using a guided tool during the registration phase in both types of systems using mapping points on the bone. The registration of image-based systems is closely linked to preoperative imaging, magnetic resonance imaging (MRI), CT, or plain radiography. Optimal component size and alignment, leg length and offset restoration, volumetric bone removal, deformity correction, and the boundaries of the bone resection are all determined preoperatively by the software using these finely detailed three-dimensional images. The surgeon can design the surgical resection plan, implant sizing, implant placing, and implant alignment before even entering the operating room if they have preoperative pictures and a preoperative plan to approve. After the joint has been exposed, this preoperative imaging is compared to the patient's anatomy during the procedure before starting the resection process. Typically, this is done using a computer-aided registry of significant and easily identifiable landmarks. The preliminary plan that the surgeon has accepted is subsequently followed by the robot. Image-based systems may have drawbacks such as higher imaging study costs, patient discomfort and extra travel for the study, and radiation risk from exposure during the CT scan. In order to generate a virtual model and surgical plan that are then carried out during the surgery, imageless systems rely on the registration of the human anatomy following surgical exposure in the operating room. The accuracy with which the surgeon enters data points at the moment of operation is the only factor that affects this registration. Implant size, location, and alignment are decided upon intraoperatively following patient registration in the absence of preoperative imaging and plan. Imageless systems have several benefits, such as lower operation costs, more patient convenience, and no radiation exposure prior to surgery. False preoperative planning and the inability to compare the anatomic registration sites after surgery to a more comprehensive set of three-dimensional images are possible drawbacks [12].

Fitted Implant Choice: Proprietary Implant vs Surgeons' Choice of Implant

Because "closed" or "open" platforms are possible with robotic systems, the surgeon may have fewer options when it comes to selecting implant systems and manufacturers of compatible implants. Certain systems have closed implant platforms, meaning that the implant(s) being utilized for the treatment can only come from one manufacturer. Different implant manufacturers and designs can be utilized on open implant platforms based on the surgeon's desire or the patient's request. For the purpose of carrying out a robotic-aided procedure, surgeons can feel uneasy using a different implant, or they might choose a particular implant design over another that is offered by the robotic system in their hospital.

Computer-Aided Navigation Systems (Navigation)

Another technology discussed in the orthopedic robotics context is computer-aided navigation systems (navigation). As a system that is passive, navigation doesn't accomplish anything for the patients. Navigation merely offers direction and information to the physician; the surgery is still carried out using traditional instruments. Navigation comes in three flavors: imageless navigation, fluoro-navigation, and CT-based navigation. The most accurate navigation is CT-based, but it requires more time and money to arrange ahead for CT images before surgery, which also exposes patients to more radiation. Imageless navigation does not make use of CT scans; instead, it relies on landmark-pointing techniques and is not sensitive to anatomical distinctiveness. While fluoroscopic navigation is useful in trauma and spine procedures, its applications in hip and knee reconstruction surgery are more constrained. Numerous studies have demonstrated the superior precision of cup alignment with navigation compared to traditional mechanical instruments, as well as the usefulness of this method for maximizing stability, limb length, and ROM. OrthoPilot, Brainlab, and the Stryker navigation system are some of the instances of navigation systems. Navigation is an independent technique that can be used either in conjunction with robotic surgery or on its own during surgical procedures. Its applications are broader and more versatile compared to robotic surgery because there are surgeries where a robot may not be necessary or applicable, whereas navigation can always be beneficial. Additionally, navigation offers a significantly wider variety of applications than current robotic technology, which mostly concentrates on hip and knee arthroplasty; treatments including the shoulder, elbow, and ankle are among them.

Current Robotic Systems

At first, robotic systems with navigation were created in the field of orthopedics to enhance clinical results and enable more accurate results to be consistently reproduced. The majority of robotic systems are made up of identical parts. The processes related to a robotically supported surgery usually include (a) developing a patient-specific system and interventional plan, (b) intraoperatively recording the model and plan to the individuals' anatomy, and (c) employing robotic support to create bone cuts and keep the preoperative plan on the patient. Though many robotic systems have been designed and prototyped, very few have been effectively applied in a medical context. The CORI surgical robot and the MAKO robotic arm interactive orthopedic system are two modern and widely utilized systems. The technology and software utilized for THA, TKA, anterior cruciate ligament (ACL) reconstruction, and high tibial osteotomy surgeries have been developed further as a result of recent breakthroughs. The ROSA knee robotic system was initially launched in Australia in 2018. In the same vein, DePuy Synthes debuted in Australia in 2021 with the VELYS robotic-assisted solution [13].

## Review

Challenges for contemporary orthopedic robotics

Studies that compared the survival rates of traditional TKA and ROBODOC after 10 years have shown similar results. Survivorship statistics, however, are currently missing for the more recent robotic system generation [14]. To maximize the safety and use of robots, surgeons and personnel must complete a substantial amount of education in addition to the costs related to robotics in the operating room. Using robotic systems may require lengthier operating times, particularly during the learning curve. Additionally, a surgeon's preferred robotic system could not work with their preferred implant system. The learning curve for a traditional TKA usually denotes the quantity of cases required to get a stable condition of outcomes. This frequently includes evaluating the surgery time reduction for robotic TKA. According to Kayani et al. [15], there were seven instances of MAKO robotically assisted TKA performed. Previous robotic systems were linked to serious difficulties. For instance, Chun et al. [16] reported a 19% complication rate during the learning phase, which included peroneal nerve injury, supracondylar fracture, rupture and dislocation of the patellar tendon, and superficial infection. These issues were avoided after doctors discovered they needed to make a larger incision. Technical issues with the previous approach necessitated an intraoperative switch to a conventional TKA in as many as 22% of instances [17,18]. These technical complications seem to be resolved with the newer-generation robots. Even with improved accuracy, existing robotic systems are programmed to follow a predetermined path. During surgery, these systems are still unable to unilaterally alter the plan in response to unforeseen circumstances or make innovative judgments. In the end, the robot will follow a strategy that it has been given based on the registration data. Image-based and imageless systems are both dependent on the quality of the data they receive, even though image-based systems might be safer because they can identify registration mistakes more quickly. As a result, while improper registration may result in the ideal plan being executed, it may also cause the plan to be executed in the wrong place, which could have disastrous consequences.

Role of AI in robotic orthopedics

Osteoarthritis Detection

AI is being used in orthopedics to detect osteoarthritis, which is typically a sign of impending joint replacement. Owing to the high frequency of osteoarthritis, machine learning (ML) has garnered significant interest in orthopedics for the purpose of automating the diagnosis and staging of osteoarthritis from imaging data. A convolutional neural network (CNN) using the Visual Geometry Group 16 (VGG-16) architecture was used by Xue et al. to automatically identify hip osteoarthritis from radiographs. Their model produced results that are similar to those of a competent doctor, with a sensitivity of 95% and a specificity of 90.7% [19]. Similarly, Tiulpin et al. used radiographs to automatically diagnose and grade knee osteoarthritis using a deep siamese CNN [20]. In addition to providing accurate diagnoses, this model also highlights the radiological characteristics that are critical to the diagnosis, providing clarity to the AI, which is now viewed as a mystery, and fostering user trust among physicians. Swiecicki et al. assessed the severity of osteoarthritis in the knee using radiographs and a Faster R-CNN model with the Kellgren-Lawrence grading system [21]. Their model showed better reproducibility and accuracy on par with a panel of radiologists.

Automated Implant Selection

One application of AI in robotics is automated implant selection for arthroplasty. Mechanical loosening, which can result from inadequate initial fixation, progressive fixation loss, biological loss from osteolysis, and/or periprosthetic infection, is a major cause of arthroplasty implant failure. Assessing implant imaging for hardware issues is crucial after joint replacement. A deep CNN was developed by Borjali et al. to recognize the mechanical loosening of total hip implants based on radiograph analysis [22]. In comparison to an experienced orthopedic surgeon, their model attained a significantly greater sensitivity of 94% and a similar specificity of 96%. They also created saliency maps to highlight the critical regions that the model utilized to diagnose, which boosted trust in the findings. Furthermore, determining the precise implant used in the initial surgery is an essential part of preoperative planning when revision surgery becomes necessary. Nonetheless, about 10% of implants are unidentifiable prior to surgery. Yi et al. addressed this problem by characterizing knee radiographs using a ResNet [23]. Their model identified differences between complete and unicompartmental knee arthroplasty, as well as between implant goods made by two distinct manufacturers. It also differed between both the existence and absence of knee arthroplasty.

Clinical Outcome Prediction

Predicting the results of surgery is another area in which ML has been used in joint reconstruction. Ramkumar et al. proposed a value-based, patient-specific payment approach in light of the particular reimbursement issues associated with joint replacement, particularly with Medicare's Comprehensive Care for Joint Replacement (CJR) bundled reimbursement model [24]. This approach makes use of preoperative outcome predicting for total joint and hip replacements. They took into account patient factors including age, ethnicity, gender, and comorbidities and used a Bayesian technique to accurately predict the duration of stay and cost after complete knee and hip arthroplasty [25]. In contrast to the CJR approach, their proposed tiered patient-specific reimbursement was intended to give a more equitable consideration of patient complexity. Using a Least Absolute Shrinkage and Selection Operator (LASSO) model, Harris et al. studied clinical outcomes and were able to predict 30-day mortality and cardiac problems after total joint replacement with a moderate degree of accuracy [26]. To enhance pre-surgical decision support, Fontana et al. focused on predicting longer-term patient-reported outcome measures (PROMs) and identifying patients at risk of not attaining meaningful gains [27]. When analyzed preoperatively, they found that all three ML algorithms, support vector machine (SVM), LASSO, and random forest, exhibited comparable fair-to-good predictive power in predicting important two-year increases in PROMs. These tools have a great deal of promise as useful tools for doctors and patients to use when making medical decisions. They can assist in reducing biases and heuristics, getting around doctors' time restrictions, offering decision support, identifying modifiable risk factors, and projecting results and problems. That being said, its use in practical situations will most likely require more accurate tools. It will also be critical to address concerns about interpretability, integration with electronic health records, continuous monitoring and validation of data, and ethical considerations.

Improving Surgeon Workflow

Improving intraoperative workflow during a robotic procedure, which can lower the learning curve and the amount of blood lost during the procedure, is one difficulty in arthroplasty. The use of patient-specific implants is becoming more common in orthopedic arthroplasty, and this could result in better results. Preoperative planning is more work with this method, though. By automating preoperative planning tailored to each patient and surgeon, Lambrechts et al. presented a novel use of ML in patient-specific joint replacement. In comparison to the manufacturer's plans, the AI-based preoperative plans were greatly improved by integrating the LASSO and SVM techniques [28]. Because of this enhancement, the surgeon had to make fewer manual corrections, which streamlined the preoperative workflow and cut down on the amount of time it took to make corrections.

Implant Research and Development

The foundation of an arthroplasty procedure is an implant. The market for arthroplasty implants is usually mature in the orthopedic medical device business, which makes it difficult for producers to differentiate their products from those of rivals in terms of therapeutic benefits. A number of recent studies have looked into the application of ML to R&D and implant optimization for total joint replacements. Eskinazi et al. optimized a deformable joint contact model utilizing a feedforward artificial neural network (ANN) to estimate thousands of loading conditions for an artificial knee implant [29]. The ANN estimated contact forces and torques with increased accuracy and over 1,000 times faster computation times than the traditional elastic foundation modeling method. This breakthrough eliminates a significant obstacle to the method's routine application in implant improvement.

Postoperative Complication Prediction

Postoperative complications represent a significant problem for any arthroplasty procedure. By providing more specific projections of anticipated adverse events, extremely accurate preoperative prognostication models could greatly improve joint decision-making and patient counseling. This is particularly important in acute situations, when there is little time to think through the possibilities. Many studies have looked into models for forecasting different elements of outcomes over the last 10 years, particularly in spine surgery. McGirt et al. employed regression analysis to forecast outcomes following lumbar surgery [30]. Age, BMI, specific symptoms, the existence of spinal abnormalities, and other predictor variables were integrated to predict clinical outcomes, including the 12-month Oswestry Disability Index (ODI), 30-day readmission rates, the requirement for rehabilitation, and return to employment. The accuracy of their model ranged from 72% to 84%. CNN and logistic regression (LR) are two models that Kim et al. developed and tested to find risk variables for postoperative complications of posterior lumbar spine fusion [31-33]. In order to forecast cardiac and wound problems, venous thromboembolism, and death, they examined data from 22,629 patients from the American College of Surgeons. When predicting all four outcome factors, CNN and LR both showed a greater area under the curve (AUC) than the American Society of Anesthesiologists categorization.

Discussion

Orthopedics and arthroplasty are starting to change as a result of robotic surgery. Robots in arthroplasty operating rooms have the potential to accomplish these goals by enhancing surgeons' ability to conduct repeatable treatments through a personalized surgical approach, originally developed to improve precision, accuracy, patient outcomes, and satisfaction rates. The benefits of robotically assisted total knee replacement, partial knee replacement, and THA have previously been demonstrated by anatomic restoration with optimum soft tissue balancing, repeatable alignment, and restoration of normal joint kinematics. Robotics allows surgeons to conduct more exact and precise surgeries consistently with a more patient-specific plan, regardless of the desired component location or limb alignment. Surgeons need to carefully assess the role of robotics in healthcare and determine where they stand on this route, much as the finest pilots who refused to change and acquire new instruments and navigation tactics eventually became obsolete when technology proved that science was safer than art. But in addition to the expense of using robotics in the operating room, a great deal of training is needed for staff members and surgeons to maximize the technology's safety and use. The return on investment is never assured, and buying a robot by itself does not guarantee better results. Operative times could be lengthier, particularly during the robotics learning curve, and a surgeon's favorite robotic system could not work well with their preferred implant system. Even with improved precision, existing robotic systems are programmed to carry out a predetermined plan; they are not capable of exercising creative judgment or unilaterally altering the plan in the middle of surgery in response to a novel circumstance. The systems now lack the ability to do soft tissue balancing, for example, in the event of a fracture necessitating cuts for an augment or a ruptured medial collateral ligament necessitating cuts for an alternative implant. While some systems are able to give the surgeon very accurate input on balancing, they are not able to modify the plan in response. Furthermore, these devices will make planned cuts no matter what they are cutting; therefore, the surgeon will need to retract the soft tissues to avoid damaging the tissues along the planned path. In order to avoid such unintentional harm, future designs will probably incorporate tracking and fail-safe measures; however, at the moment, robots are unable to distinguish between different types of tissue. Lastly, using the registration data that it has been given, the robot will cut in accordance with a plan. Image-based and imageless systems are both dependent on the quality of the data they receive, even though image-based systems might be safer because they can identify registration mistakes more quickly. Consequently, inaccurate registration may result in the ideal plan being carried out, but it may also cause the plan to be carried out in the entirely wrong place, which could have disastrous consequences.

## Conclusions

Although it initially required longer operative periods, robots in arthroplasty have so far improved consistency and decreased variability, and there is growing evidence that suggests better clinical outcomes. With the advent of AI, robotics has the potential to be a useful tool for surgeons in the future as they optimize patient-specific arthroplasty. One thing is certain: robotics is probably here to stay, even though more research is required to clearly determine its costs and benefits. Significant progress in the field of surgical orthopedics has been made possible by the integration of AI into robotic surgery procedures. Through customized surgical approaches, these technologies aim to increase the surgeon's ability to perform repeatable procedures with more accuracy, precision, and better patient outcomes. AI is currently being used in robotic surgery to improve radiographic outcomes for total joint arthroplasty by decreasing outliers and increasing accuracy. But soon, with developments in AI and ML, robotic arthroplasty should be commonplace for a larger number of patients.
